# Relationship between vitamin D status and the inflammatory state in patients with chronic spontaneous urticaria

**DOI:** 10.1186/1476-9255-11-2

**Published:** 2014-02-03

**Authors:** Alicja Grzanka, Edyta Machura, Bogdan Mazur, Maciej Misiolek, Jerzy Jochem, Jacek Kasperski, Alicja Kasperska-Zajac

**Affiliations:** 1 Department of Internal Diseases, Dermatology and Allergology in Zabrze, Medical University of Silesia in Katowice, M. Curie-Skłodowskiej 10, 41-800 Zabrze, Poland; 2 Department of Pediatric in Zabrze, Medical University of Silesia in Katowice, Katowice, Poland; 3 Department of Microbiology and Immunology in Zabrze, Medical University of Silesia in Katowice, Katowice, Poland; 4 Clinical Department of Otolaryngology in Zabrze, Medical University of Silesia in Katowice, Katowice, Poland; 5 Department of Basic Medical Sciences in Bytom, Medical University of Silesia in Katowice, Katowice, Poland; 6 Department of Prosthetic Dentistry in Bytom, Medical University of Silesia in Katowice, Katowice, Poland

**Keywords:** Vitamin D, 25-hydroxyvitamin D, Chronic spontaneous urticaria (CSU), Acute phase response, C-reactive protein

## Abstract

**Background:**

Chronic spontaneous urticaria (CSU) is an immune-inflammatory disease, characterized by acute phase response (APR) and immune activation. There has been increasing evidence showing that vitamin D deficiency/insufficiency is associated with increased incidence and/or severity of immune-inflammatory disorders.

**Aim:**

To assess relationship between vitamin D status and C-reactive protein (CRP), a nonspecific inflammatory marker of CSU activity.

**Methods:**

Concentrations of CRP and 25-hydroxyvitamin D [25(OH)D], a biomarker of vitamin D status were measured in serum of CSU patients and compared with the healthy controls.

**Results:**

Serum 25(OH)D concentration was significantly lower in CSU group as compared with the normal subjects. The prevalence of vitamin D deficiency (< 20 ng/ml) was significantly higher in patients with CSU than among normal population. There were no significant differences in prevalence of 25(OH)D insufficiency between the groups. Serum CRP concentrations were significantly higher in CSU patients as compared with the healthy subjects. There were no significant correlations between CRP and 25(OH)D concentrations in CSU patients.

**Conclusions:**

CSU is associated with lower serum 25(OH)D concentration and higher prevalence of its deficiency. The results failed to show any effect of vitamin D status on circulating CRP concentrations in CSU. A potential role of vitamin D in pathogenesis and/or additive therapy of CSU needs to be examined in other cohorts of CSU patients as well as in larger studies.

## Introduction

Chronic spontaneous urticaria (CSU) is an inflammatory disease, characterized by acute phase response (APR) and in many cases by the immune-activation. C-reactive protein (CRP) is a marker of systemic CSU activity, reflecting the systemic effects of inflammatory mediators associated with the disease, including IL-6 [1-5]. The altered function of the neuro-endocrine-immune system has also been recognized in the pathogenesis [6].

There has been increasing evidence showing that vitamin D deficiency/insufficiency is associated with increased incidence and severity/activity of the immune-inflammatory disorders. Vitamin D has immunomodulatory properties and is able to suppress the inflammatory milieu, including IL-6 and CRP synthesis [7-9]. In the clinical practice, vitamin D status is assessed by measurement of the circulating level of 25-hydroxyvitamin D [25(OH)D], considered as the best indicator of vitamin D status, including its availability [10,11].

Little data are available on vitamin D status in CSU patients. To estimate the prevalence of vitamin D deficiency and insufficiency in CU, serum 25(OH)D concentrations were compared between CSU patients and the healthy controls. In addition, relationship between vitamin D status and C-reactive protein (CRP), a nonspecific inflammatory marker of the disease activity, was assessed.

## Materials and methods

35 patients with active CSU (20 and 15 men women; median age: 35 years, range: 22-51) with a median disease duration of 3.5 years were enrolled in the study.

In all cases, any known causes of CSU were ruled out by appropriate investigations. Each patient underwent the following tests: routine laboratory tests (full blood count, urine analysis, ESR, C-reactive protein, serum glucose, hepatic functions, and creatinine), stool (for parasites), hepatitis serology, antinuclear and antithyroid microsomal antibodies, thyroid function tests, chest X-ray and abdominal ultrasonography. Additionally, dental, and ENT consultations as well as the autologous serum skin test (ASST) were performed.

All the patients were divided into several subgroups, according to the urticarial activity score (UAS), autologous serum skin test (ASST), glucocorticoids therapy response as well as serum 25(OH)D concentration defined as: a) sufficiency (≥30 ng/ml), b) insufficiency (between 20 and 29 ng/ml), c) deficiency (< 20 ng/ml), d) critically low level (< 10 ng/ml),

UAS according to EAACI/GALEN/EDF guidelines [12] was estimated during four days and on the blood sampling day and graded as follows: mild (0–8), moderate (9–16) and severe (17–24). The study comprised 12 patients with mild and 23 patients with moderate-severe utricaria symptoms.

H1- antihistamine drugs were withdrawn at least 4 days before blood sampling. At the time of blood sampling, only 9 patients were receiving low doses of oral glucocorticoids (GC) (5–10 mg prednisolone per day). None of the remaining patients had been taking immunosuppressants or any other drugs, for at least 8 weeks before the study.

The control group comprised 33 sex-, age- and BMI (< 30) matched the healthy subjects.

The Ethics Committee of the Medical University of Silesia approved of the study and written, informed consent was obtained from all the subjects participating.

### Blood collection

All blood samples were obtained between 7 and 9 a.m. by anticubital puncture. As the circulating levels of 25(OH)D vary depending on the season, the concentration was evaluated in summer (June through September).

### Assay of 25(OH)D

Serum 25(OH)D concentration was measured with the use of an automated direct electrochemiluminescence immunoassay (Elecsys, Roche Diagnostic, Mannheim Germany) with the detection limit of 3.0 ng/ml. Sufficient vitamin D concentration was defined as ≥30 ng/ml.

### Assay of CRP

Serum CRP concentration was measured by the turbidimetric latex agglutination method (CRP-Latex, BioSystems SA, Barcelona, Spain) with a detection limit of 1.0 mg/l. Elevated serum CRP was defined as higher than 5.0 mg/l.

### Autologous serum skin test (ASST)

Intradermal ASST was performed following the method by Sabroe et al. [13]. Serum-induced a red wheal response of diameter greater by at least 1.5 mm than that of a control wheal induced by physiological saline was accepted as positive. Skin prick test with histamine served as a positive control.

### Statistical analysis

Results are expressed as median and inter-quartile ranges. Because data were not distributed normally, nonparametric tests were used. Kruskal-Wallis variance analysis was used to screen differences between the groups. Mann-Whitney *U* and the Fisher exact tests were used to compare data between the patient groups and the normal population. Spearman’s rank test was used for correlations. The probability value of *P* < .05 was assumed significant.

## Results

### Serum 25(OH)D concentration

**Figure 1:**
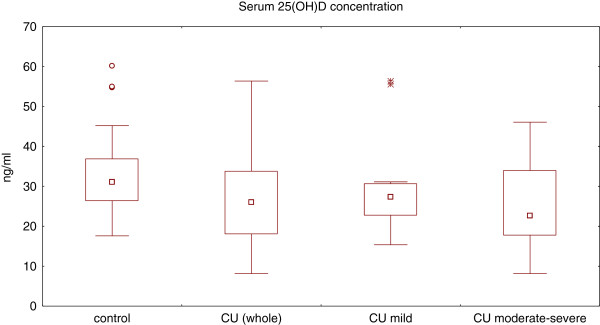
**Serum 25(OH) concentration in healthy subjects and chronic spontaneous urticaria (CSU) patients with different disease activity.** CSU (whole) vs. control, p < 0.05; CSU-moderate/severe vs. control, p < 0.05; CSU-moderate/severe vs. CSU-mild, p > 0.05; CSU-mild vs. control, p > 0.05.

Serum 25(OH)D concentration was significantly lower in CSU group as compared with the normal subjects (median: 26.0 *vs.* 31.1 ng/ml, **p = 0.017**) (Figure 1). There were no significant differences in serum 25(OH)D concentration between the CSU patients with mild and moderate-severe symptoms (median: 27.3 *vs.* 22.6 ng/ml, p = 0.53). However, 25(OH)D concentrations were slightly yet significantly lower in moderate-severe CSU than those of the controls (22.6 *vs*. 31.1 ng/ml, **p = 0.048**). There were no significant differences in concentrations between mild CSU patients and the healthy subjects (27.3 *vs.* 31.1 ng/ml, p = 0.13) (Figure 1).

Moreover, there were no significant differences in serum concentrations of 25(OH)D in moderate-severe CSU patients with and without glucocorticoid therapy (p = 0.57).

CSU subgroup without glucocorticoids was significantly lower as compared with the healthy subjects (data not shown).

### Deficiency and insufficiency of vitamin D

The prevalence of vitamin D deficiency (< 20 ng/ml) was significantly higher in patients with CU than in the normal population. There were no significant differences in the prevalence of 25(OH)D insufficiency (≥20 ng/ml but <30 ng/ml) between CU patients and the normal subjects (Table 1).

**Table 1:** Vitamin D status in CSU patients as compared with healthy subjects

**Serum 25(OH)D**	**CSU patients**	**Healthy subjects**	**p-value**
**concentration**	**n = 35**	**n = 33**	
**Insufficiency**	11/35	13/33	p = 0.41
(≥20 ng/ml but <30 ng/ml)	(31.4%)	(39.4%)	
**Deficiency**	11/35	2/33	p=0.025*
(<20 ng/ml)	(31.4%)	(6%)	
**Severe**			
**deficiency**	1/35	0/33	p = 0.52
(<10 ng/ml)	(2.9%)	(0%)	

*25(OH)D* - 25-hydroxyvitamin D; *CSU* - chronic spontaneous urticaria; *statistically significant difference (p<0.05).

### Serum CRP concentration

Serum CRP concentrations were significantly higher in CSU patients (currently untreated with GC) as compared with the healthy subjects (median: 7.1 *vs.* 0.8 mg/l, p < 0.0001). There was significant difference in CRP serum concentration between patients with mild and moderate-severe CSU (without GC) (median: 1.7 *vs.* 11.7 g/L, p < 0.0001). CRP serum concentration was significantly lower in moderate-severe CU patients with GC as compared with those without GC therapy, and was similar to the healthy subjects (median: 1.8 *vs.* 11.7 *vs.* 0.8 g/L, p < 0.5).

No significant differences in 25(OH)D and CRP concentrations between ASST(+) and ASST(-) CU patients were observed.

### Associations

There were no significant correlations between serum concentrations of CRP and 25(OH)D (r = -0.16, p = 0.45) in CSU patients without GC therapy. In addition, no correlation was found between the disease duration and 25(OH)D concentration.

## Discussion

To the best of our knowledge, there have only been two reports regarding vitamin D in CU [14,15].

Thorp et al. reported that serum 25(OH)D concentration was reduced as compared to that in patients with allergic rhinitis [14]. However, it has been suggested that the prevalence of severe vitamin D deficiency was significantly higher in patients with allergic rhinitis than among the normal population [16]. Therefore, in the present study we compared CSU patients with the healthy subjects. Similarly to the previous study, serum 25(OH)D concentration in CSU patients decreased as compared with the healthy subjects.

Thorp et al. showed that the proportion of all subjects with vitamin D deficiency (defined as 25-OHD < 30 ng/ml) was not significantly different between the 2 groups: chronic urticaria, 48% (12/25) versus controls, 28% (7/25; p = 0.24) [14]. In our study the subjects were divided into three groups according to serum 25(OH)D concentration, to describe vitamin D status: deficiency (<20 ng/ml), insufficiency (between 21 and 29 ng/ml), critically low level (< 10 ng/ml) as defined by most experts [10,17]. In contrasts to the previous study the proportion of vitamin D deficiency proved significantly higher in CSU patients.

The reason for such discrepancy is unclear, although it may be related to various factors, including 1) differences in the control group (healthy versus allergic rhinitis subjects), 2) seasons during which blood was collected, 3) regions and/or countries, 4) lifestyle. It has been reported that serum 25(OH)D concentrations in the European countries are lower than in the United States [10,17,18].

There were not significant differences in serum 25(OH)D concentrations in CSU patients with and without glucocorticoid therapy. We found no association between serum concentrations of 25-(OH)D and the glucocorticoids doses in CSU patients. This is in accordance with the previous studies indicating that serum 25(OH)D concentration is unaffected by treatment, even with high doses of glucocorticoids [19]. Taken together, it seems unlikely that the glucocorticoid treatment of our patients is responsible for lower serum concentrations of 25(OH)D.

Although our and Thorp et al. results point to some changes in vitamin D status, such as a decrease in serum 25(OH)D concentration [14], conclusions regarding the reasons for reduced concentration as well as relevance of such results for a possible risk factor in CSU and/or the disease activity cannot yet be drawn. While some data suggest that 25(OH)D levels are associated with an increased activity/severity of the inflammatory diseases [7], we found no relationship between serum concentrations of 25(OH)D and CRP - a marker of CSU activity [20].

Lower 25(OH)D concentration may therefore appear as a mere secondary phenomenon, manifested as a response to different stimuli, including inflammation, and as such, may not contribute in any way to pathogenesis of the disease. As chronic urticaria may be associated with lower serum 25OHD concentration during the active period of the disease, it would be interesting to recognize whether such phenomenon is present shortly after the disease onset and after a long lasting remission, suggesting vitamin D deficiency as a possible risk factor for CSU.

Interestingly, it has been reported that vitamin D metabolites regulate the synthesis of matrix metalloproteinases (MMP) [21] and vitamin D insufficiency is associated with the increased circulating levels of MMP9 and CRP [22], suggesting a possible mechanism for tissue damage in chronic inflammatory conditions, including CHD and diabetes [22]. On the other hand, it is known that both MMP9 and CRP concentrations are elevated in CSU [23]. In our study we did not observe any significant association between concentrations of 25(OH)D and CRP. Associations between vitamin D status and MMP9 overproduction in CSU should be established.

The limitations of this study included small sample size and single assessment of 25 (OH)D concentration performed in summer. Because serum 25(OH)D concentrations are lower in winter, it should be interesting to compare the seasonal differences. Therefore, we cannot exclude that prevalence of 25(OH)D deficiency might be higher following the summer season.

The serum 25(OH)D concentrations vary extensively between studies and depend on different environmental factors. Serum 25(OH)D concentration ≥ 30 ng/ml is defined by most experts as optimal vitamin D status with respect to maintenance of mineral homeostasis. So far, immune-inflammatory consequences of vitamin D insufficiency/deficiency, assessed on the basis of 25(OH)D concentrations alone, are unclear [17,18,24].

### Clinical implications

Current data demonstrate the importance of screening for vitamin D deficiency measured by serum concentration of 25(OH)D in CSU patients. In addition, such observations may have certain therapeutic implications. Interestingly, it has been demonstrated that in patients suffering from idiopathic chronic urticaria, isolated pruritus, and rash with low 25(OH)D level, the symptoms resolution is often possible with oral supplementation of vitamin D [15,25].

We speculate that treatment of vitamin D deficiency would not only preserve mineral homeostasis but, due to possible immunomodulatory and anti-inflammatory effects of vitamin D, might have a beneficial impact on CSU activity. Vitamin D supplementation may provide an important and viable complement to the already existing CSU therapy. Higher doses of corticosteroids may be required in therapy of patients with the immune-inflammatory diseases and concomitant vitamin D insufficiency/deficiency [26]. In addition, the association was found between low serum 25(OH)D concentration and the cardiovascular mortality [27].

Interestingly, it has been demonstrated that vitamin D insufficiency/deficiency promotes immune-inflammatory response and exogenous vitamin D is able to diminish activation of APR [9,24].

It seems important to know whether the anti-inflammatory effects of exogenous vitamin D influence CSU activity.

## Conclusions

CSU is associated with lower serum 25(OH)D concentration and higher prevalence of its deficiency. The results failed to show any effect of vitamin D status on the circulating CRP concentrations in CSU. Taking into consideration that increased vitamin D intakes might reduce the incidence and/or severity of immune-inflammatory disorders, the potential role of vitamin D in etiopathogenesis and/or treatment of CSU calls for examination in other cohorts of CSU patients as well as in larger studies.

## Abbreviations

CSU: Chronic spontaneous urticaria; APR: Acute phase response; CRP: C-reactive protein; 25(OH)D: 25-hydroxyvitamin D; ASST: Autologous serum skin test; UAS: Urticaria activity score; GC: Glucocorticoids; MMP: Matrix metalloproteinases; ENT: Ear, nose, throat.

## Competing interests

The authors declare that they have no competing interests.

## Authors’ contributions

AG: conceived and designed the study, collected samples and provided clinical data, contributed to data analysis and interpretation and wrote the manuscript. EM: collected samples and provided clinical data, wrote the manuscript BM: performed the lab analysis, contributed to data analysis and interpretation. MM: provided clinical data (ENT consultations), identified patients and reviewed the manuscript. AKZ: conceived, designed and supervised the study as well as reviewed the manuscript. JJ: contributed to statistical data analysis and reviewed the manuscript. All authors read and approved the final manuscript. JK: provided clinical data (dental consultations) and identified patients.
